# Multidisciplinary-derived clinical score for accurate prediction of long-term mortality in fibrotic lung disease patients

**DOI:** 10.1186/s40001-024-01644-7

**Published:** 2024-01-20

**Authors:** Yu-Wan Liao, Yi-Ming Chen, Ming-Cheng Liu, Yu-Cheng Wu, Chiann-Yi Hsu, Pin-Kuei Fu, Wen-Nan Huang, Yi-Hsing Chen

**Affiliations:** 1 Integrated Care Center of Interstitial Lung Disease, Taichung Veterans General Hospital, Taichung, 40705 Taiwan; 2 Division of Allergy, Immunology and Rheumatology, Department of Internal Medicine, Division of Allergy, Taichung Veterans General Hospital, Taichung, 40705 Taiwan; 3 Division of Translation Medicine, Department of Medical Research, Taichung Veterans General Hospital, Taichung, 40705 Taiwan; 4 Department of Radiology, Taichung Veterans General Hospital, Taichung, 40705 Taiwan; 5 Department of Critical Care Medicine, Taichung Veterans General Hospital, Taichung, 40705 Taiwan; 6 Biostatistics Task Force, Department of Medical Research, Taichung Veterans General Hospital, Taichung, 40705 Taiwan; 7 Division of Clinical Research, Department of Medical Research, Taichung Veterans General Hospital, Taichung, 40705 Taiwan; 8 Department of Post-Baccalaureate Medicine, College of Medicine, National Chung Hsing University, Taichung, 40200 Taiwan; 9 Department of Medical Research, Taichung Veterans General Hospital, 1650 Taiwan Boulevard Sect. 4, Taichung, 407219 Taiwan

**Keywords:** Idiopathic pulmonary fibrosis, Interstitial lung disease, Multidisciplinary discussion, 6-min walk test

## Abstract

**Background:**

Idiopathic pulmonary fibrosis (IPF) stands out as one of the most aggressive forms of interstitial lung diseases (ILDs), currently without a definitive cure. Multidisciplinary discussion (MDD) is now considered a cornerstone in diagnosing and differentiating ILD subtypes. The Gender-Age-Physiology (GAP) score, developed to assess IPF prognosis based on sex, age, forced vital capacity, and diffusion capacity for carbon monoxide (DLCO), is limited in not considering dyspnea and functional impairment during the walking test. We proposed a MDD-based clinical score for mortality prediction among those patients.

**Methods:**

From December 2018 to December 2019, we enrolled ILD patients with IPF and non-IPF and followed-up them till December 2020. Based on DLCO, modified Medical Research Council (mMRC) Dyspnea Scale, and six-minute walking test (6MWT) distance, a functional score was developed for mortality prediction.

**Results:**

We enrolled 104 ILD patients, 12 (11.5%) died by the one-year follow-up. In receiver operating characteristic (ROC) curve analysis, DLCO (% predicted) was the most accurate variable predicting one-year mortality with an area under curve (AUC) of 0.88 (95% confidence interval [CI] = 0.80–0.94), followed by mMRC Dyspnea Score (AUC = 0.82 [95% CI = 0.73–0.89]), 6MWT distance (AUC = 0.80 [95% CI = 0.71–0.88]), and GAP score (AUC = 0.77 [95% CI = 0.67–0.84]). Only the GAP score (hazard ratio [HR] = 1.55, 95% CI = 1.03–2.34, *p* = 0.0.37) and functional score (HR = 3.45, 95% CI = 1.11–10.73, *p* = 0.032) were significantly associated with one-year mortality in multivariable analysis.

**Conclusion:**

The clinical score composite of DLCO, mMRC Dyspnea Scale, and 6MWT distance could provide an accurate prediction for long-term mortality in ILD patients, laying out a helpful tool for managing and following these patients.

**Supplementary Information:**

The online version contains supplementary material available at 10.1186/s40001-024-01644-7.

## Background

Idiopathic pulmonary fibrosis (IPF) stands out as one of the most aggressive forms of interstitial lung diseases (ILDs), currently without a definitive cure [1]. IPF manifests as an undue proliferation of fibroblasts, which in turn produce an excessive amount of extracellular proteins, notably collagen [2]. This overproduction facilitates recurrent tissue scarring and fibrosis within the lung parenchyma, culminating in increased rigidity of the lungs. Consequently, this rigidity impairs oxygen absorption and gas exchange capabilities [2]. Post-diagnosis, the prognosis for IPF is grim, often resulting in fatality within 2–3 years [3]. The etiology remains elusive, and there are no established treatments that offer significant improvement in patient outcomes [4]. Furthermore, even the term IPF itself is still debatable. While some researchers urge “splitting” it into distinct subcategories that respond uniquely to specific treatments, others suggest "lumping" IPF together with other types of advancing fibrotic lung diseases that have common underlying causes and exhibit similar disease progression [5].

Recent advancements in the field have underscored the importance of novel predictors of outcomes in fibrotic lung diseases. Notably, emerging studies and review articles have highlighted the significance of the PD-1/PD-L1 axis and mediastinal lymphadenopathy as novel markers in the progression and prognosis of these diseases[6, 7]. These developments indicate a shift towards more precision-based approaches in understanding and managing ILD.

Owing to the wide pathological and prognostic diversity of ILD disorders with a paradoxical clinical and radiological mimicry, a multidisciplinary discussion (MDD) among experts, including pulmonologists, chest radiologists, pathologists, and rheumatologists, is pivotal in making an accurate ILD diagnosis. This pivotal role was highlighted by the European Respiratory Society (ERS) and American Thoracic Society (ATS) since 2001 [8], and the published practice guidelines and epidemiologic studies over the recent years emphasized the necessity and importance of MDDs in managing ILDs [9, 10]. When compared to diagnoses made by individual physicians, this technique boosts diagnostic confidence and interobserver agreement among ILD specialists [11, 12].

Several prospective registries for ILDs have been created in North America and Europe [13–15]. Studies based on well-designed and well-performed patient registries can provide a real-world perspective on clinical practice, patient outcomes, safety, and clinical comparative and support cost-effectiveness analyses. They study the disease course in compliance with current diagnostic and treatment guidelines and can serve as important tools for decision-making [16–18]. However, there have been few published epidemiological studies on ILD, particularly in Asia [19]. In addition, many registries have focused only on IPF with limited data for other fibrotic ILD subtypes (CTD-ILD or non-IPF populations) [20]. The Gender-Age-Physiology (GAP) score, modified Medical Research Council (mMRC) Dyspnea Scale, ventilatory efficiency slope, six-minute walking test (6MWT) distance, DLCO, and CO2 partial pressure in the arterial blood at maximal exercise could be important predictors for mortality [21–23]. Since the landmark SENSCIS [24] and INBUILD [25] trials demonstrated that systemic sclerosis-associated ILD, CTD-ILD, and other non-IPF progressive fibrosing ILDs could benefit from the same antifibrotic treatment as IPF, registries of patients with and without IPF are warranted. Moreover, identifying prognosis factors among all fibrotic lung diseases is critical for patients and clinicians to introduce antifibrotic agents and start the rehabilitation program in the early stage of fibrotic lung disease [26, 27].

The Registry of Interstitial Lung Disease (REGILD) is a prospective, single-center registry study enrolling IPF and non-IPF populations in central Taiwan. Every patient enrolled in the REGILD registry is evaluated by MDD experts, including pulmonologists, rheumatologists, radiologists, and pathologists. This study aimed to identify associated prognostic factors in the REGILD cohort and develop a new clinical score for predicting long-term prognosis in real world. Moreover, it compared the overall mortality prediction power of the newly developed score to the standard GAP score, based on factors estimated during the first year of follow-up.

## Methods

### Study design, patient enrollment, and ethics

The REGILD is a prospective, single-center registry study enrolling IPF and non-IPF populations in the Integrated Care Center of Interstitial Lung Disease of Taichung Veterans General Hospital, a tertiary referral center in Taiwan. Between December 2018 and December 2019, we enrolled patients with documented fibrotic lung diseases on high-resolution computed tomography (HRCT) that were confirmed by an MDD team comprising expert pulmonologists, rheumatologists, radiologists, and pathologists. Patients enrolled into analyzed were followed-up at least 1-year till December 2020. IPF patients were categorized as “definite,” “probable,” or “indeterminate” with IPF according to the American Thoracic Society/European Respiratory Society/Japanese Respiratory Society/Latin American Thoracic Association guidelines. ILD patients with established diagnoses of connective tissue diseases were classified as CTD-ILD. Patients classified as having interstitial pneumonia of autoimmune features (IPAF) must meet the IPAF classification criteria [14]. The exclusion criteria were age < 20 years and HIV infection. All enrolled patients provided written informed consent. This study was conducted in compliance with the Declaration of Helsinki and approved by the Ethics Committee of Taichung Veterans General Hospital (IRB number: CE18325B; date of approval: December 18, 2018).

### ILD assessment protocol in the REGILD registry cohort

The index day was defined as the day the patient signed the informed consent form. The participants completed the mMRC Dyspnea Scale Dyspnea Questionnaire on the index day. Within one week of enrolment, the participants were administered the pulmonary function test (PFT), 6MWT, and cardiopulmonary exercise test. Baseline demographic data, including age, Gender, and ethnicity, were recorded. Clinical data comprised presenting symptoms, physical examination, environmental and occupational exposures, smoking history, and comorbidities. The GAP score was calculated for each patient.

### PFT and 6MWT procedure

Forced vital capacity (FVC) and DLCO were obtained from spirometry results according to the recommendations of the American Thoracic Society [28]. The 6MWT was performed according to the guidelines of the American Thoracic Society [29]. The patients were instructed to walk as far as possible in six minutes in a corridor between two orange traffic cones placed 30 m apart. Data on oxygen saturation and the distance walked in six minutes were obtained.

### Statistical analysis

Descriptive analysis was performed with categorical data presented as absolute numbers and relative frequencies. Continuous variables are presented as median (interquartile range [IQR]) for non-parametric data. The Mann–Whitney U test was used to compare continuous variables. The Chi-square and Fisher’s exact tests were used to compare categorical variables. P-value was adjusted using Benjamini–Hochberg correction when comparing survival and non-survival group to control the false discovery rate [30]. A Cox proportional regression was used to examine possible factors for mortality with univariate and multivariate models. The area under the receiver operating characteristic (ROC) curve (AUC) indicates the predictive value. Better predictive power was acknowledged when the AUC was > 0.70. Kaplan–Meier estimates and log-rank tests were used to calculate mortality based on the following three variables: DLCO, mMRC Dyspnea Scale, and 6MWT distance. Statistical analyses were performed using SPSS Statistics (version 22; IBM Corporation, Armonk, NY, USA). A *p*-value < 0.05 was considered statistically significant.

## Results

### Baseline characteristics and pulmonary physiology

Table 1 shows patient characteristics in this registry. One hundred and four participants were enrolled: 33 with (31.7%) and 71 without (68.3%) IPF. Compared with patients without IPF, patients with IPF were older and more often male. In our registry, among patients without IPF, the most common CTD was idiopathic inflammatory myositis (35.5%), followed by primary systemic sclerosis (29.0%) and IPAF (12.7%). The proportion with definite usual interstitial pneumonia (UIP) patterns on HRCT scans was higher in the IPF (81.8%) than in the non-IPF (47.9%) group. The median GAP score was 3 in the IPF group (IQR = 2–4.5), compared to 2 in the non-IPF group (IQR = 1–3). mMRC dyspnea scale were higher in the IPF group than in the non-IPF group. The proportion of patients receiving antifibrotic agents was higher in the IPF group.

**Table 1:** Study characteristics among IPF and CTD-ILD patients. One hundred and four participants were enrolled in the study, with 33 (31.7%) having idiopathic pulmonary fibrosis (IPF) and 71 (68.3%) without. Patients with IPF were typically older, more often male, and had higher definite usual interstitial pneumonia (UIP) patterns on HRCT scans compared to those without IPF. Additionally, the IPF group had higher median GAP scores and Modified Medical Research Council (mMRC) dyspnea scale readings, and a greater proportion were receiving antifibrotic agents

	IPF (n = 33)^a^	Non-IPF (n = 71)^a^	*p*-value^b^
Age	69 (62–74)	61 (51–66)	< 0.001
Male	24 (72.7%)	16 (22.5%)	< 0.001
Smoking status			
No	13 (39.4%)	57 (80.3%)	< 0.001
Current smoker	2 (6.1%)	3 (4.2%)	
Ex-smoker	18 (54.5%)	11 (15.5%)	
Smoking index	42.5 (21.3–77.5)	30 (18.8–65)	0.381
Charlson Comorbidity Index (CCI)	3.0 (1–4)	2.00 (2–4)	0.749
Pattern in HRCT			
NSIP	0 (%)	37 (52.1%)	< 0.001
UIP pattern (Probable UIP)	6 (18.2%)	24 (33.8%)	
UIP pattern (Definite UIP)	27 (81.8%)	10 (14.1%)	
GAP score	3 (2–4.5)	2 (1–3)	< 0.001
mMRC Dyspnea Scale	1 (1–3)	1 (0–1)	0.014
Anti-fibrotic agents	20 (60.6%)	12 (16.9%)	< 0.001
Pulmonary function test			
FVC (L)	2.2 (1.8–2.8)	2.0 (1.7–2.6)	0.451
FVC (% predicted)	80.0 (61–96)	74.0 (60–88)	0.550
FEV1 (% predicted)	81.0 (64–91.5)	77.0 (59–87)	0.287
FEV1/FVC (%)	84.0 (78.5–89.5)	82.0 (78–85)	0.033
DL_CO_ (% predicted)	64.0 (39–79)	71.0 (53.5–82.5)	0.112
6MWT			
Distance (m)	403.5 (345–462)	461 (399–516.75)	0.007
Initial SpO2	96.0 (93.8–97)	96.0 (95–97)	0.168
SpO_2_ after 6MWT	88.0 (83.3–92)	89.5 (85–92)	0.365
Nadir SpO2 < 90%	18.0 (60.0%)	32.0 (50.0%)	0.494
One- year Mortality	4 (12.1%)	8 (11.3%)	1.000

^a^Median (IQR); n (%)

^b^Mann-Whitney *U* test, Chi-Square test, Fisher’s exact test

*IPF* idiopathic pulmonary fibrosis *UIP* usual interstitial pneumonia, *HRCT* high-resolution computed tomography, *NSIP* non-specific interstitial pneumonia, *GAP* gender, age, physiology, *mMRC* modified Medical Research Council, *FVC* forced vital capacity, *FEV1* forced expiratory volume in one second, *DLCO* diffusion capacity for carbon monoxide, *6MWT* 6-min walking test, *SpO*_*2*_ oxygen saturation

In this cohort, baseline FVC, FEV1, and DLCO (% predicted) data did not differ significantly between groups. Nevertheless, IPF patients had a higher FEV1/FVC (84.0%, *p* = 0.033). On the contrary, the non-IPF group had greater 6MWT distances than the IPF group (461 m vs 403.5 m, *p* = 0.007). Oxygen saturations (SpO_2_) before and after the 6MWT did not differ significantly between the IPF and non-IPF groups (*p* = 0.365). More than half of the participants in both groups had a nadir SpO_2_ of < 90% (Table 1).

### Primary outcome of one-year mortality

Of the 104 patients, 12 died before the cutoff in December 2020. Eight of them died from pneumonia and respiratory failure, three succumbed to malignancies (two to lung cancer and one to colon cancer), and one patient died from an ischemic stroke. Five deceased patients had IPF, and seven had non-IPF (Table 2). Patients in the mortality group had significantly higher GAP and mMRC Dyspnea Scores (all *p* < 0.01). In addition, patients in the mortality group had a lower % predicted FEV1 (56.0% vs 78.5%, *p* = 0.031), lower % predicted DLCO (39.5% vs71.5%, *p* < 0.001), and lower initial SpO2 (92 [IQR = 90.8–96] vs 96 [IQR = 05–97], *p* = 0.007). In contrast, patients in the survival group could walk further in the 6MWT (451.5 m vs 307.5 m, *p* = 0.014) and were less desaturated afterward (89.5% vs 78.5%, *p* = 0.020; Table 2).

**Table 2:** Study characteristics among survival and non-survival groups. Out of the 104 patients, 12 died before the December 2020 cutoff, with eight succumbing to pneumonia and respiratory failure, three to malignancies, and one to an ischemic stroke. Of the deceased, five had idiopathic pulmonary fibrosis (IPF) and seven did not. The mortality group had significantly higher GAP and mMRC Dyspnea Scores, along with lower predicted FEV1, DLCO percentages, and initial SpO2, compared to the survival group

	Survival (n = 92)^a^	Mortality (n = 12)^a^	*p*-value^b,c^
Age	62 (57.3–69.8)	67.5 (61.3–70.5)	0.301
Male	32 (34.8%)	8 (66.7%)	0.055
Classification of ILD			
IPF	28 (30.4%)	5 (41.7%)	0.513
Non-IPF	64 (69.6%)	7 (58.3%)	
Pattern in HRCT			
NSIP	41 (44.6%)	2 (16.7%)	0.048
UIP pattern (Probable UIP)	41 (44.6%)	10 (83.3%)	
UIP pattern (Definite UIP)	10 (10.9%)	0 (0.0%)	
GAP score	2 (1–3)	3 (3–5)	0.002
mMRC Dyspnea Scale	1 (0–1)	3 (2–3)	< 0.001
Antifibrotic agents	29 (31.5%)	3 (25.0%)	0.751
Pulmonary function test			
FVC (L)	2.2 (1.8–2.7)	1.5 (1.3–2.5)	0.087
FVC (% predicted)	77.0 (64.3–88.8)	57.0 (47.8–88.8)	0.127
FEV1 (% predicted)	78.5 (66.0–89.0)	56 (49.0–79.8)	0.031
FEV1/FVC (%)	83.0 (78.3–86.0)	82 (71.8–90.0)	0.636
DLCO (% predicted)	71.5 (56.0–82.8)	39.5 (31.0–53.5)	< 0.001
6MWT			
Distance (m)	451.5 (386.3–507.0)	307.5 (159.5–409.5)	0.014
Initial SpO_2_	96. (95.0–97.0)	92.0 (90.8–96.0)	0.007
SpO_2_ after 6MWT	89.5 (85.3–92.0)	78.5 (73.5–86.3)	0.002
Nadir SpO_2_ < 90%	44 (50.0%)	6 (100%)	0.028

^a^Median (IQR); n (%)

^b^Mann-Whitney U test, Chi-Square test, Fisher’s exact test

^c^Adusted p-value using Benjamini–Hochberg correction to control the false discovery rate

*ILD* Interstitial lung disease, *IPF* idiopathic pulmonary fibrosis, *UIP* usual interstitial pneumonia, *HRCT* high-resolution computed tomography, *NSIP* non-specific interstitial pneumonia, *GAP* gender, age, physiology, *mMRC* modified Medical Research Council, *FVC* forced vital capacity, *DLCO* diffusion capacity for carbon monoxide, *6MWT* 6-min walking test, *SpO*_*2*_ oxygen saturation

### Predictive factors for one-year mortality

**Fig. 1:**
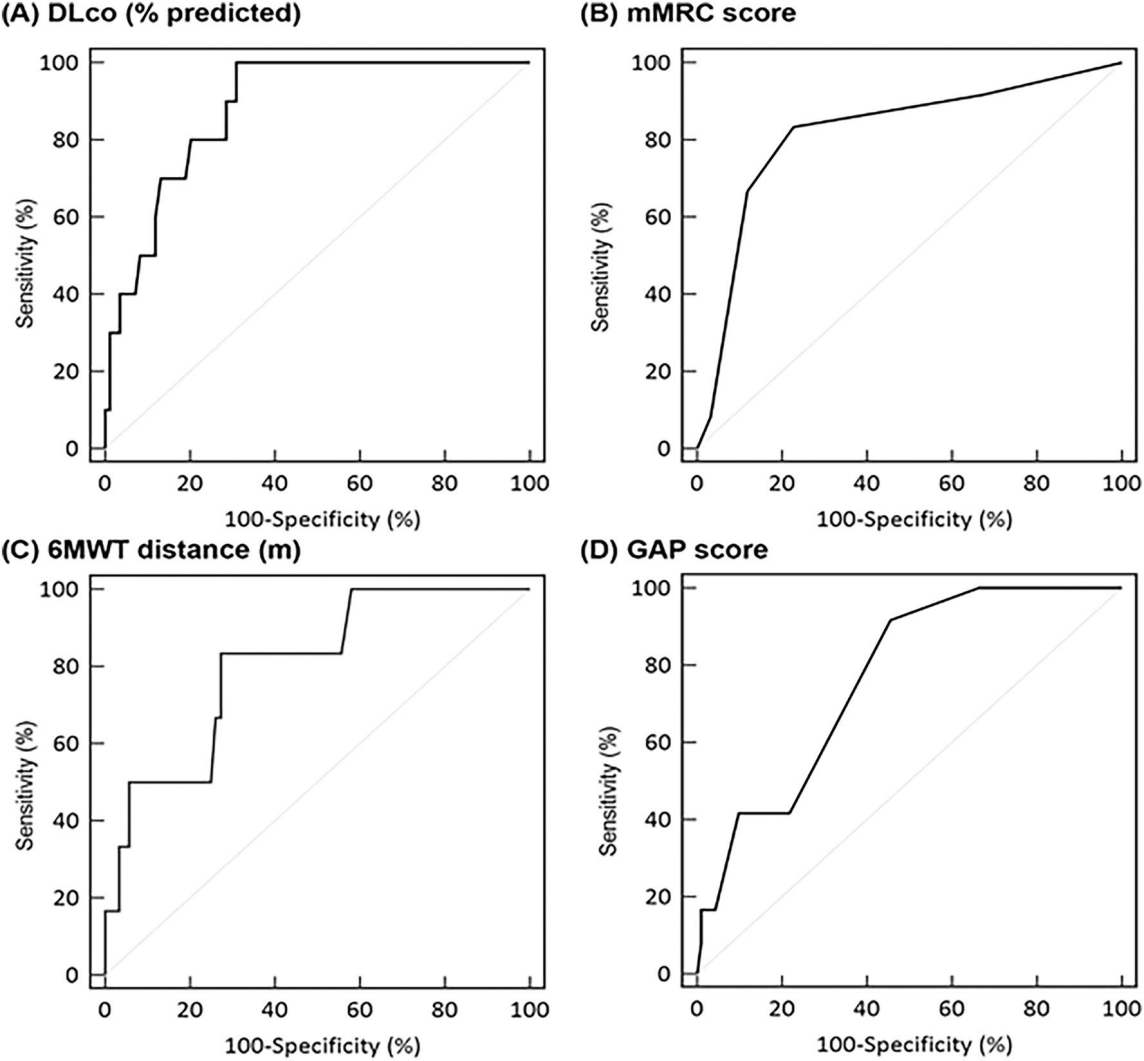
ROC curves: **A** DLCO (%predicted); **B** mMRC score; **C** 6MWT distance (m); **D** GAP score

ROC curve analysis was performed to evaluate the efficacy of DLCO (% predicted), mMRC Dyspnea Score, and 6MWT distance, in addition to GAP score for predicting one-year mortality in this cohort (Fig. 1). The AUCs, optimal cutoff values, and other statistical indicators are presented in Additional file 1: Table S1. DLCO (% predicted) was the most accurate variable with an AUC of 0.88 (95% confidence interval [CI] = 0.80–0.94), followed by mMRC Dyspnea Score (AUC = 0.82 [95% CI = 0.73–0.89]), 6MWT distance (AUC = 0.80 [95% CI = 0.71–0.88]), andGAP score (AUC = 0.77 [95% CI = 0.67–0.84]). The cutoffs were 63 for DLCO (% predicted), 1 for mMRC Dyspnea Score, 392 m for 6MWT distance, and 2 for GAP score (Additional file 1: Table S1).

**Fig. 2:**
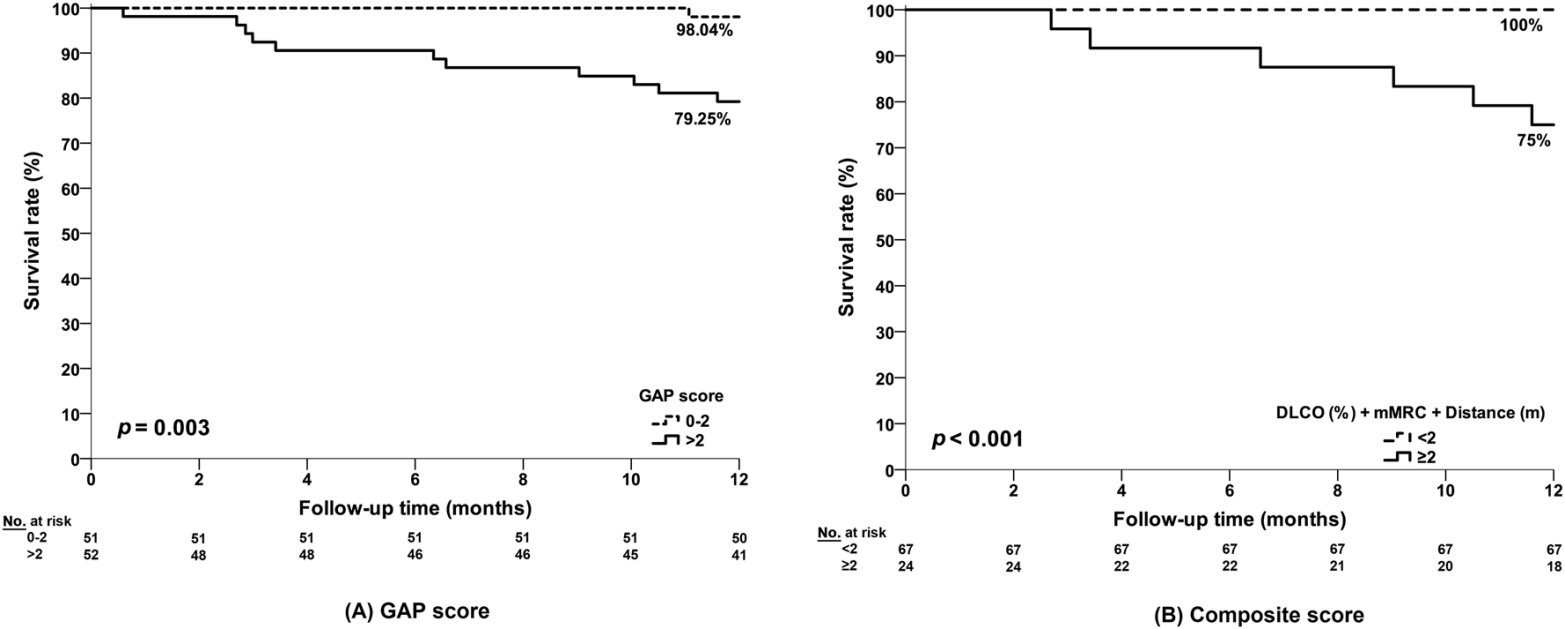
Kaplan–Meier Survival curves: **A** According to the GAP score (score 0–2 vs score > 2); **B** According to the composite score (score < 2 vs score ≥ 2)

We proposed a clinical scoring system based on DLCO (% predicted), mMRC Dyspnea score and 6MWT distance, where One point is scored if any of the following variables are fulfilled: (1) DLCO < 63% predicted, (2) mMRC Dyspnea Score > 1, (3) or 6MWT distance < 392 m, with a minimum score of 0 and maximum score of 3. Figure 2 delineates Kaplan–Meier curves for GAP score and the proposed composite score. Patients with GAP score ≤ 2 had a significantly higher survival rate compared to those with GAP score > 2 (98.04% vs 79.25%, *p* = 0.003; Fig. 2a). Likewise, patients with composite score < 2 had significantly higher survival rate than those with composite score ≥ 2 (100% vs 75%, p < 0.001; Fig. 2b).

### Univariate and multivariate analyses of factors associated with one-year mortality

Univariate and multivariate Cox analyses for one-year mortality are shown in Table 3. Higher GAP scores were associated with decreased survival in the univariate (HR = 1.55, 95% CI = 1.19–2.01, *p* = 0.001) and multivariate (HR = 1.55 [95% CI = 1.03–2.34], *p* = 0.037) models. Likewise, the clinical composite score (DLCO + mMRC score + 6MWT distance) was associated with higher risk of mortality in the univariate (HR = 4.46 [95% CI = 1.72–11.58], *p* = 0.002) and multivariate (HR = 3.45 [95% CI = 1.11–10.73], *p* = 0.032) models. Other factors including age, gender, UIP pattern, antifibrotic agents, and pulmonary function were not significant predictors of mortality in the univariate analysis.

**Table 3:** Univariate and multivariate analyses of factors associated with one-year mortality. Higher GAP scores were linked to reduced survival in both univariate and multivariate models, with respective hazard ratios (HR) of 1.55 (p = 0.001) and 1.55 (p = 0.037). Similarly, a clinical composite score combining DLCO, mMRC score, and 6MWT distance was associated with an increased mortality risk in both univariate and multivariate analyses. Other factors such as age, gender, UIP pattern, antifibrotic agents, and pulmonary function did not significantly predict mortality in the univariate analysis

	Simple model		Adjusted for GAP score	
	HR (95%CI)^a^	*p*-value	HR (95%CI)^a^	*p*-value
Demographics				
Age	1.02 (0.97–1.07)	0.495		
Male	3.28 (0.99–10.91)	0.052		
CTD-ILD	0.66 (0.21–2.07)	0.475		
Antifibrotic agents	0.73 (0.20–2.71)	0.643		
Pulmonary function				
FVC (% predicted)	0.99 (0.96–1.02)	0.439		
FEV1 (%)	0.99 (0.96–1.02)	0.547		
FEV1/FVC (%)	0.97 (0.90–1.06)	0.498		
GAP score	1.55 (1.19–2.01)	0.001	1.55 (1.03–2.34)	0.037
DLCO (%) + mMRC score + 6MWT distance (m)	4.46 (1.72–11.58)	0.002	3.45 (1.11–10.73)	0.032

^a^HR (95% CI) from the Cox regression

## Discussion

In the Oldham et al. cohort, approximately 70% of patients who met IPAF criteria had HRCT or pathological UIP, whereas the Chartrand et al. [31], Ahmad et al. [32] and Kelly et al. [33] cohorts had HRCT or pathological NSIP/OP/NSIP with OP patterns in more than 60% of patients. In this prospective cohort study, IPAF patients had a higher proportion of UIP. However, this is not the case for all IPAF patients. This variability underscores the need for individualized diagnostic pathways and the importance of a thorough clinical, radiological, and histopathological assessment to accurately classify and treat each case.

In this study, we established the predictive value of DLCO, mMRC Dyspnea Scale, and 6MWT distance for one-year mortality in patients with newly diagnosed fibrotic lung disease, including both IPF and non-IPF populations, in a tertiary referral center in Taiwan. Utilizing an MDD approach, we further categorized patients into IPF or non-IPF groups after a comprehensive evaluation using these tests. Significantly, the composite score from these assessments emerged as a crucial tool in identifying the specific type of lung disease in each patient. This level of diagnostic precision is vital for guiding physicians in selecting the most appropriate preventative and therapeutic interventions, thereby optimizing treatment outcomes. The findings from this study underscore the importance of a holistic, multidisciplinary assessment in effectively managing fibrotic lung diseases and underscore the value of these specific measures in predicting patient outcomes and informing clinical decision-making.

The 2022 ATS/ERS/JRS/ALAT official guidelines recommend a one-year interval for assessing the progression of Interstitial Lung Disease (ILD), highlighting its importance in identifying progressive pulmonary fibrosis (PPF) phenotypes. Our study, while not specifically focused on PPF, proposes that mortality within one year of an ILD diagnosis indicates a more severe progression, leading us to select one-year mortality as our primary endpoint [34].

Significantly, in our cohort, the one-year mortality rate was 12%, with no discernible difference between IPF and non-IPF groups. This observation differs from a study conducted by Moor CC et al., 2021, which identified a one-year all-cause mortality rate of 20% among IPF patients, notably higher than the rate observed in our cohort [35]. The variance in mortality rates may be attributed to the inclusion of patients in more advanced stages of IPF with elevated GAP stages and significantly diminished lung function (FVC < 20% of predicted value) in the study by Moor CC et al. [35]. In contrast, our study primarily involved newly diagnosed ILD patients who exhibited relatively better-preserved lung function. This study thus provides a real-world insight into the one-year survival of patients with newly diagnosed ILD, emphasizing the importance of early-stage identification and intervention.

The findings of this study are particularly relevant in the context of evolving research that underscores the significance of novel predictors of outcomes in fibrotic lung diseases. Recent studies and review articles have emphasized the emerging role of the PD-1/PD-L1 axis and mediastinal lymphadenopathy as novel markers, offering potential insights into the progression and prognosis of these conditions [6, 7]. This highlights the growing importance of precision medicine approaches in understanding and managing ILD.

Our findings reveal a notable correlation between DLCO and the 6MWT distance, suggesting a comprehensive approach to assessing both pulmonary function and physical capability. This correlation is crucial as it provides a broader perspective on patient health, extending beyond basic pulmonary function tests. Patients with advanced fibrosis often exhibit a reduced DLCO and cover shorter distances in the 6-min walk test (6MWT). A study on diffuse systemic sclerosis-associated ILD, which included 121 patients, demonstrated a notable correlation between SpO2 levels during the 6MWT and DLCO [36]. Additionally, we came across a study examining the correlation between the 6-min walk distance (6MWD) and DLCO in patients with COPD. This study identified a correlation coefficient of 0.39 (p < 0.05) between 6MWD and DLCO [37]. Furthermore, a 2017 study investigated the relationship between 6MWD and DLCO in ILD patients [38]. Their results showed a positive correlation, with a Pearson correlation coefficient of 0.272 (p = 0.001).

In our cohort, the classification of IPAF within CTD-ILD aligns with current diagnostic understanding. While IPAF represents a unique subset, its inclusion in CTD-ILD reflects the complexities and overlaps within ILD classifications. We also explored the HRCT imaging characteristics of non-IPF patients, noting significant variability among different ethnic groups. This diversity highlights the importance of personalized approaches in diagnosing and managing ILD, particularly in diverse populations.

On the univariate and multivariate analysis of factors associated with one-year mortality, we found that the GAP score and the newly established predictor composite of DLCO, mMRC Dyspnea Scale, and 6MWT distance were the only significant predictors for one-year mortality. This aligns with the most recent ATS/ERS/JRS/ALAT clinical practice guideline, which defines a clinically suspected case of IPF as a patient with unexplained patterns of bilateral pulmonary fibrosis on chest radiography or chest computed tomography, bibasilar inspiratory crackles, and age > 60 years [34]. The guideline also emphasizes the role of MDD in diagnosing IPF, highlighting the importance of multidisciplinary approaches in accurate diagnosis and management.

Our study's findings resonate with those of Shou et al., who demonstrated that static DLCO and changes in DLCO are predictive of mortality (95% CI = 0.08–0.90) [39]. Similarly, Goh et al. found that DLCO alone or in combination with FVC predicts mortality in Interstitial Lung Disease [40], and Walsh et al. reported an association of DLCO with increased mortality in patients with connective tissue disease-related fibrotic lung disease (CTD-FLD; p = 0.013, 95% CI = 0.95–0.99) [41].

Furthermore, we observed that the mMRC Dyspnea Scale (AUC = 0.82) can predict one-year mortality in fibrotic lung disease patients. This finding is consistent with Cheng et al.'s study, where the mMRC Dyspnea Scale (AUC = 0.87) was used to predict mortality in IPF patients [21]. Nishiyama et al. also found it to be a reliable predictor of survival in IPF patients (HR = 2.402, 95% CI = 1.495–3.858, p = 0.0003) [42].

The 6MWT distance (AUC = 0.80) also predicted one-year mortality in fibrotic lung disease patients, with a cutoff point at 392 m. This is in line with the findings of Cheng et al., where non-survivors experienced more oxygen desaturation during the test [21], and Mancuzo et al.'s report that a 6MWD of < 330 m or < 70% of the predicted value is linked to a substantially decreased survival rate in IPF patients [22].

Patients in the mortality group had significantly higher GAP scores. The median GAP score was 3 in the IPF group, compared with 2 in the non-IPF group, paralleling the findings of Cheng et al., where a GAP score ≥ 4 was associated with 1-year mortality in IPF patients [21]. Notably, the HR of our new predictor composite of DLCO, mMRC Dyspnea Scale, and 6MWT distance was higher than that of the GAP score (HR = 3.45 [1.11–10.73] vs HR = 1.55 [1.03–2.34]), indicating its potential utility in clinical practice. Furthermore, the effectiveness of the GAP score and the potential for enhanced accuracy with modified GAP scores tailored for specific populations are areas warranting further exploration [43].

## Conclusion

In this study, we focused on confirming the mortality predictive role of a multidisciplinary discussion (MDD) clinical score, comprising DLCO, mMRC Dyspnea Scale, and 6MWT distance, among newly diagnosed fibrotic lung disease patients within Asian populations. Our findings, demonstrating an adjusted hazard ratio (HR) comparable to that of the GAP score in the multivariate analysis, underscore the potential utility of this composite score in clinical practice. While we successfully identified significant predictors for one-year mortality, it's important to acknowledge the limitation of our relatively small sample size. Despite this, our study contributes valuable insights, particularly for managing both IPF and non-IPF patients and highlights the need for further research in diverse populations to refine and validate our findings. This effort to develop and validate novel predictive tools reflects an ongoing commitment to improving patient care and outcomes in the field of interstitial lung disease.

### Supplementary Information

**Additional file 1.** **Table S1.** The AUC and cutoff for different predictors. DLCO (% predicted) was the most accurate variable for predicting outcomes, with an Area Under the Curve (AUC) of 0.88, followed by mMRC Dyspnea Score (AUC = 0.82), 6MWT distance (AUC = 0.80), and GAP score (AUC = 0.77). The respective cutoffs for these variables were 63% for DLCO, 1 for mMRC Dyspnea Score, 392 meters for 6MWT distance, and 2 for GAP score.

## Data Availability

The datasets used and/or analyzed during the current study are available from the corresponding author on reasonable request.

## Abbreviations

6MWT: Six-minute walking test
6MWD: Six-minute walking distance
ATS: American Thoracic Society
CI: Confidence interval
CTD-ILD: Connective tissue disease-associated interstitial lung disease
DLCO: Diffusion capacity for carbon monoxide
ERS: European Respiratory Society
FVC: Forced vital capacity
GAP: Gender-Age-Physiology
HR: Hazard ratio
IPAF: Interstitial pneumonia of autoimmune features
IPF: Idiopathic pulmonary fibrosis
IQR: Interquartile range
MDD: Multidisciplinary discussion
mMRC: Modified Medical Research Council
PFT: Pulmonary function test
REGILD: Registry of Interstitial Lung Disease
ROC: Receiver operating characteristic
