# Supplementary material for: Multidisciplinary-derived clinical score for accurate prediction of long-term mortality in fibrotic lung disease patients
Source: Eur J Med Res. 2024 Jan 20;29:69. doi: 10.1186/s40001-024-01644-7 (PMC10799536; doi:10.1186/s40001-024-01644-7)
Supplement: Supplementary file 1 — Additional file 1. Table S1. The AUC and cutoff for different predictors. DLCO (% predicted) was the most accurate variable for predicting outcomes, with an Area Under the Curve (AUC) of 0.88, followed by mMRC Dyspnea Score (AUC = 0.82), 6MWT distance (AUC = 0.80), and GAP score (AUC = 0.77). The respective cutoffs for these variables were 63% for DLCO, 1 for mMRC Dyspnea Score, 392 meters for 6MWT distance, and 2 for GAP score. [file 40001_2024_1644_MOESM1_ESM.docx]

**Table S1.** The AUC and cutoff for different predictors

|  | **AUC** | **(95%CI)** | ***p*-value** | **Cut point** | **Sensitivity** | **Specificity** | **PPV** | **NPV** | **Accuracy** |
| --- | --- | --- | --- | --- | --- | --- | --- | --- | --- |
| **DLCO (%)** | 0.88 | (0.80-0.94) | <0.0001 | ≤63 | 100 | 69.05 | 27.78 | 100 | 72.34 |
| **mMRC Dyspnea Scale** | 0.82 | (0.73-0.89) | <0.0001 | >2 | 83.33 | 77.17 | 32.26 | 97.26 | 77.88 |
| **6MWT distance (m)** | 0.80 | (0.71-0.88) | 0.001** | ≤392 | 83.33 | 72.73 | 17.24 | 98.46 | 73.40 |
| **GAP score** | 0.77 | (0.67-0.84) | <0.0001 | >2 | 91.67 | 54.35 | 20.75 | 98.04 | 58.65 |

The cutoff values were determined by the area under the receiver operating characteristic (ROC) curve (AUC).
